# First Swedish case of fatal equine parasitic encephalitis by *Halicephalobus gingivalis*

**DOI:** 10.1186/s13028-023-00719-5

**Published:** 2023-12-15

**Authors:** Karin Maria Olofsson, Norbert van de Velde, Simone Peletto, Barbara Iulini, Laura Pratley, Behzad Modabberzadeh, Emilian Małek, Giulio Grandi

**Affiliations:** 1 Department of Pathology and Wildlife Diseases, National Veterinary Institute (SVA), Uppsala, Sweden; 2 Istituto Zooprofilattico Sperimentale del Piemonte, Liguria e Valle D’Aosta, Turin, Italy; 3 Evidensia Specialisthästsjukhuset Helsingborg, Helsingborg, Sweden; 4 Farm & Animal Health, Kävlinge, Sweden; 5 Department of Microbiology, National Veterinary Institute (SVA), Uppsala, Sweden; 6 Department of Biomedical Sciences and Veterinary Public Health, Swedish University of Agricultural Sciences (SLU), Uppsala, Sweden

**Keywords:** Encephalitic and nephritic nematodiasis, Horse, Phylogenetic analysis, Zoonosis

## Abstract

**Background:**

*Halicephalobus gingivalis* is a nematode with zoonotic potential which can cause fatal opportunistic infections in various mammals. The parasite has never been diagnosed in Sweden, in any species, prior to the presented case.

**Case presentation:**

An imported 21-year-old Icelandic mare developed severe neurological signs. The horse was eventually euthanized and submitted for post-mortem examination where severe lesions in the kidneys were noted. Histopathology revealed the presence of *H. gingivalis* in both kidneys and the brain. Phylogenetic analysis of the parasite determined it to belong to Lineage 1.

**Conclusions:**

With the occurrence of *H. gingivalis* in Sweden, the disease should be added to the list of differential diagnoses in cases with acute onset of neurological disease in both horses and other mammals including humans.

## Background

*Halicephalobus gingivalis* is a saprophytic rhabditic nematode which can cause opportunistic, predominantly fatal, infections in mammals. Cases have mostly been reported in equids, including zebras, but even in cattle and humans [1, 2]. Equine cases have been reported throughout the world with reports from several European countries [2]. However, no previous cases have been reported in Sweden. The exact life cycle of this parasite is not fully understood, nor is the full pathogenesis within the host. As the parasite is free-living in soil, the suggested routes of infection are soil contamination of ulcers in the skin or oral mucosa or through inhalation. This proposed route is strengthened by the notion that the first description of the parasite was from an equine oral granuloma [3]. The presence of viable nematodes within blood vessels suggests a hematogenous migration through the host, as well as suggesting migration through the lymphatics [4, 5]. The main target sites of this parasite are brain and kidney with occasional lesions in other organs. The clinical neurological signs develop quickly and include ataxia and other unspecific neurological signs. Urinary conditions such as stranguria and hematuria have been suggested to be induced by tissue damage by the invading parasite [6]. Most cases of *H. gingivalis* infections are diagnosed at post-mortem examination, independent of species affected, where gross lesions of the brain can be mild or absent. Histological changes together with rhabditic nematodes are diagnostic, but confirmation via ancillary testing such as PCR is suggested especially if limited tissue samples are available. Nadler et al. [7] identified four different lineages of *H. gingivalis*. Subsequent genetic analysis of *H. gingivalis* isolates of the current case could give insights into which lineage the current isolates belong to, adding to the knowledge on genetic variation and geographic distribution.

## Case presentation

A 21-year-old Icelandic mare, imported from Iceland to Sweden five years earlier (2016), presented to the equine hospital (Evidensia Specialisthästsjukhuset, Helsingborg, Sweden) with a two-day history of vague clinical signs that included haematuria, pyrexia (38.8 °C), icteric gingival mucous membranes and mild, intermittent ataxia. The mare had received parenteral benzyl-penicillin (15 mg/kg), gentamicin (6.6 mg/kg) and flunixin meglumine (0.5 mg/kg) the previous day and the day of presentation to the equine hospital. One year earlier, the horse had an episode of haematuria without fever or ataxia and inflammation with a dilated renal pelvis in the left kidney, discovered during ultrasound examination (Evidensia Specialisthästsjukhuset, Helsingborg). The horse responded well to treatment with penicillin and gentamicin and clinical signs resolved fully.

**Fig. 1:**
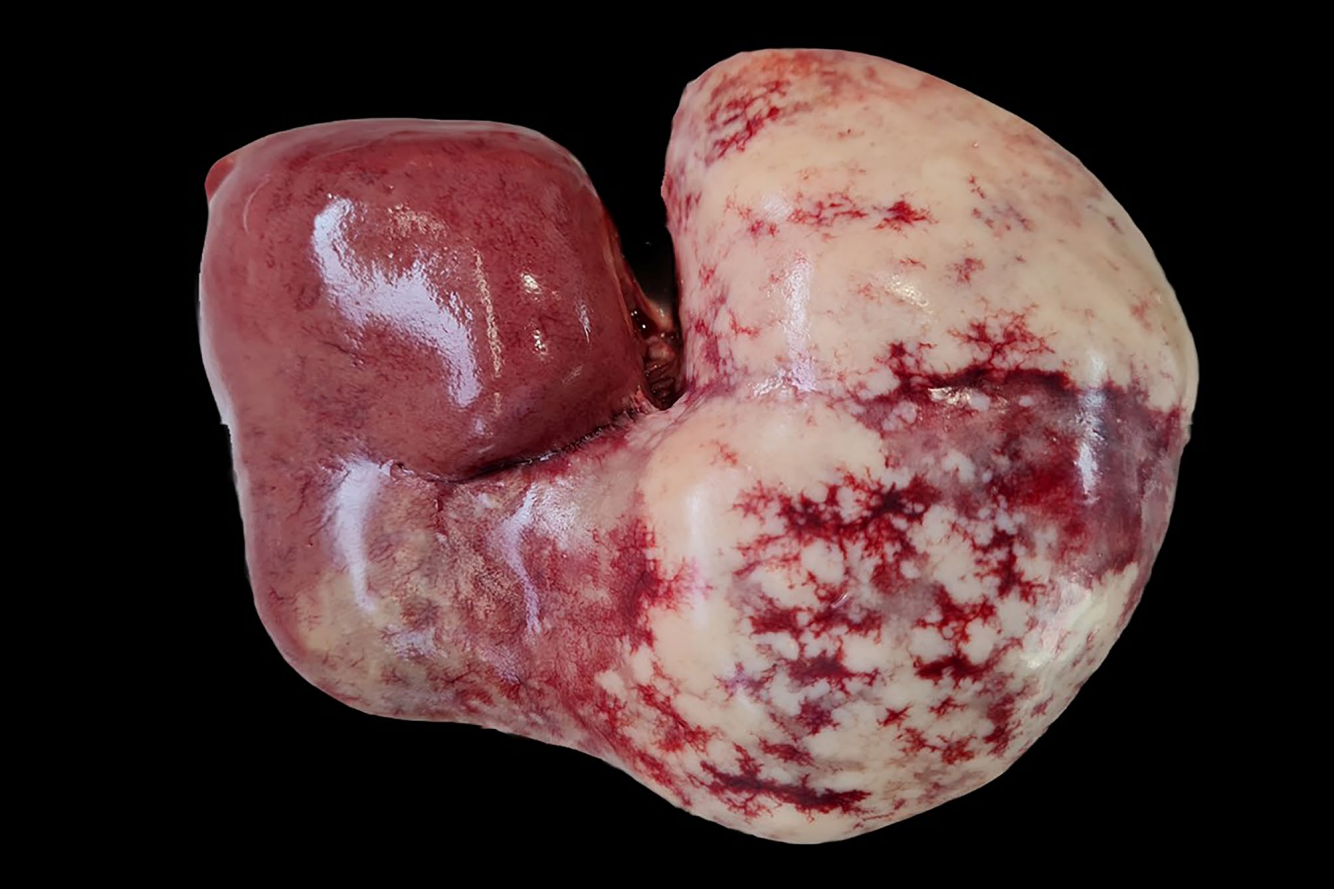
Right kidney at necropsy. The cranial half of the right kidney was markedly enlarged with dense white tissue containing several haemorrhages, effacing the normal renal tissue

**Fig. 2:**
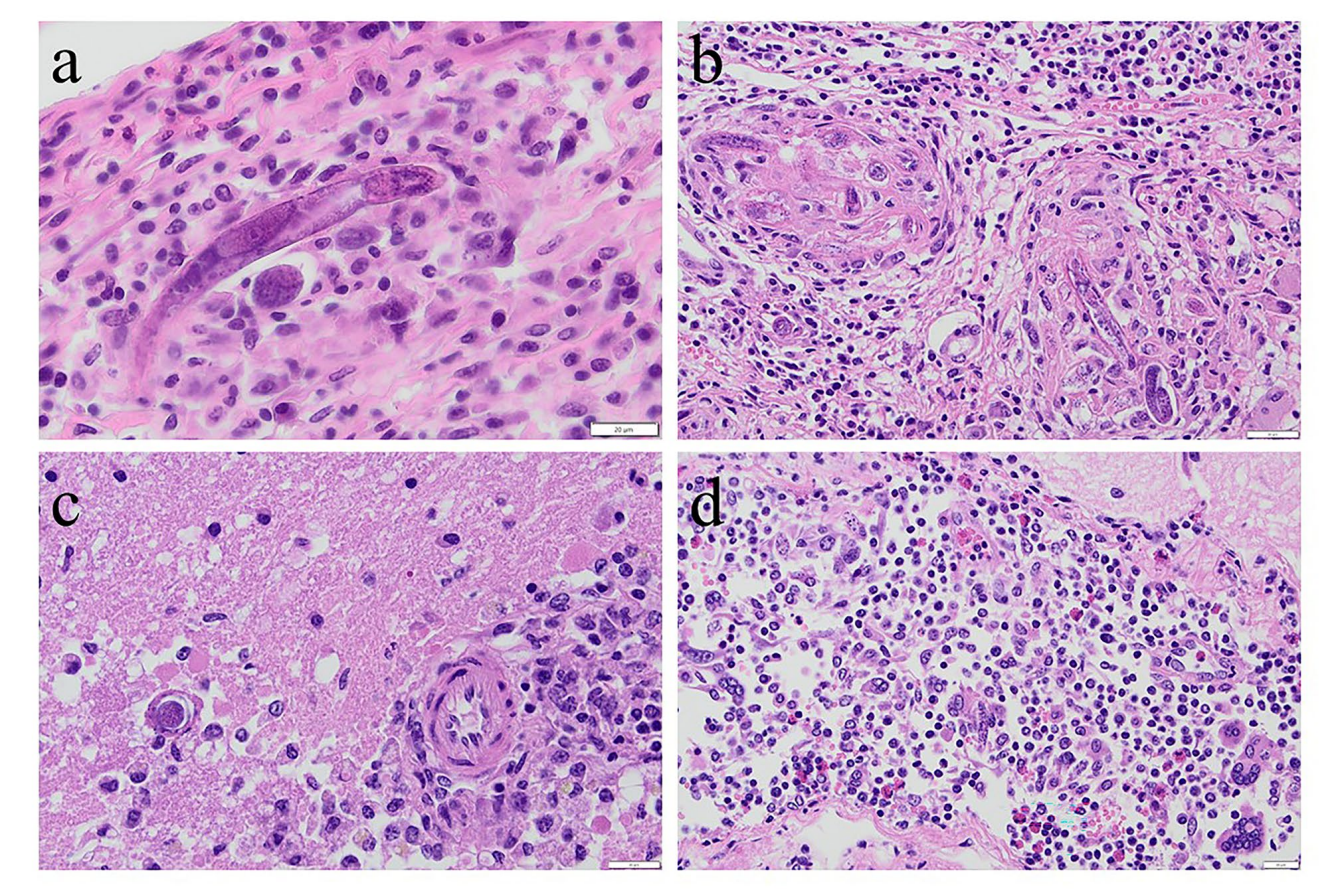
Histological lesions with intralesional *Halicephalobus gingivalis*. **a**. In the renal parenchyma, intralesional nematodes displayed a smooth cuticle and in transversally sectioned parasites a rhabditic esophagus was noted. H&E, bar 20 μm. **b**. The dense white tissue of the right kidney was composed of a severe granulomatous nephritis with numerous intralesional rhabditic nematodes. H&E, bar 20 μm. **c**. Intravascular larvae were noted in the cerebral parenchyma with mild lymphocytic to eosinophilic encephalitis. H&E, bar 20 μm. **d**. The leptomeninges of the cerebellum was moderately to severely expanded by eosinophils, and mononuclear cells including giant cells. Scattered throughout the tissue, and partly digested in the cytoplasm of giant cells, were nematode larvae. H&E, bar 20 μm

On submission to the equine hospital the horse was mildly disorientated and ataxic but not pyrexic. Mild hyponatremia (131,9 × 10^9^/L – normal range: 132–144 × 10^9^/L), mild anaemia (haemoglobin of 9,4 g/dL – normal range: 10,7–16,5 g/dL) and mild lymphopenia (1,17 × 10^9^ g/dL – normal rage: 1,5–5,1 × 10^9^ g/dL), moderate hypercreatininemia (167 µmol/L – normal range: 0,0-141 µmol/L) were noted during blood analysis. No urinalysis was performed. Lactate and glucose levels were within normal limits. Upon subsequent clinical examination, it was found that the horse had a grade 2 ataxia and hypermetria in both thoracic and pelvic limbs, which exacerbated on a small circle. Additionally, mild ptosis in the left eye was found with other cranial nerves functioning normally. Reduced proprioception in front legs, weakness to the left on both static and dynamic tail pull was noted. During the following four hours the neurological signs progressed significantly, the horse had become obtunded, started head pressing, had poor anal tone and fell spontaneously, corresponding with grade 4 ataxia on the Mayhew scale [8]. Evaluation of the clinical neurological signs indicated involvement of forebrain, brainstem, and spinal cord with both upper and lower motor neuron deficits. Further cranial nerve deficits developed with aggravated ptosis and facial paralysis on the left side, bilateral prolonged pupillary light reflex and reduced muscular tone of the tongue. Swabs from the upper respiratory tract were taken for routine PCR-analysis for equine herpesvirus (EHV) type 1 and 4, and equine influenza virus (EIV) H3N8, all of which were negative. No improvement in clinical signs was seen after treatment with high dose intravenous dexamethasone (0.12 mg/kg). Subsequently, it was decided to euthanize the horse and submit it for a routine post-mortem examination (Farm & Animal Health, Kristianstad, Sweden). Post-mortem examination revealed that the cranial half of the right kidney was markedly enlarged with dense white tissue containing several haemorrhages, effacing the normal renal tissue (Fig. 1). The mucosa of the urinary bladder was generally thickened and haemorrhagic. No gross abnormalities were noted in the brain. Based on the clinical and post-mortem findings, formalin-fixed samples of the spleen, liver, kidney, and brain were submitted for further histological evaluation (SVA, Uppsala, Sweden). Tissue sections were routinely processed and stained with haematoxylin and eosin (HE stain). During histological examination it was noted that the renal tissue was almost completely replaced by large infiltrates of lymphocytes, plasma cells, macrophages, and rare eosinophils. Within this inflammation multiple poorly delineated granulomas were present. Mostly within the centre of these granulomas, numerous nematodes were identified (Fig. 2b). These nematodes were approximately 10–25 μm in diameter with a smooth cuticle, a pseudocoelomic cavity filled with 2–3 μm basophilic structures and a rhabditic oesophagus (Fig. 2a). Within the sections of the brain, fewer similar nematodes were seen and were mostly perivascular associated with mild granulomatous and eosinophilic inflammation (Fig. 2c). The leptomeninges were thickened by multifocal infiltrates of lymphocytes, macrophages, eosinophils, and multinucleated giant cells with intracytoplasmatic remnants of parasites (Fig. 2d). Based on the morphology of the nematodes and their location within brain and kidney, they were suggested to be *H. gingivalis*.

**Fig. 3:**
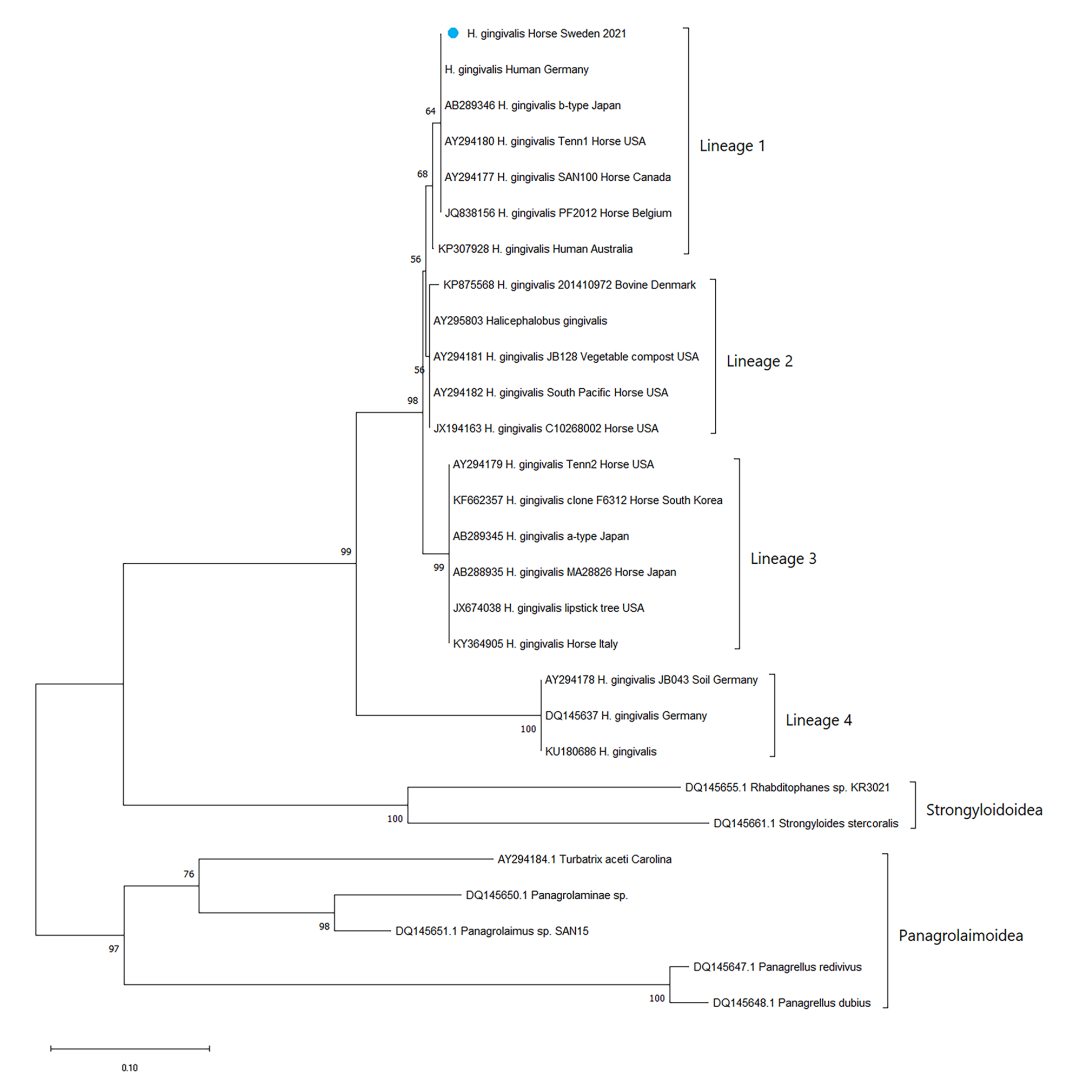
Maximum-likelihood (ML) tree for 21 *H. gingivalis* isolates and 7 outgroup sequences. Phylogeny was inferred by an alignment of 216 nucleotides. The phylogenetic tree includes the novel Swedish *H. gingivalis* isolate (GenBank acc. no. OQ834433, blue dot) and homologous sequences available in GenBank. Accession numbers, host and country of origin are indicated for each sequence, when available. Bootstrap (1,000 replicates) values over 50 are shown at the internal nodes. The length of each pair of branches represents the distance between sequence pairs. The scale bar represents the percentage of nucleotide differences

Paraffin blocks were forwarded for genetic analysis of the *H. gingivalis* 28 S rRNA gene (Istituto Zooprofilattico Sperimentale del Piemonte Liguria e Valle d’Aosta, Turin, Italy). DNA was extracted from brain tissue in the paraffin blocks using the QIAamp DNA FFPE Advanced kit (Qiagen) and submitted to PCR amplification of a fragment of the 28 S ribosomal rRNA gene using the forward primer #504, 5′-CAA GTA CCG TGA GGG AAA GTT G-3′, and reverse primer #503, 5′-CCT TGG TCC GTG TTT CAA GAC G-3′, according to Nadler et al. [7]. The resulting 262 bp (bp) PCR amplicon was then purified and sequenced on a 16-capillary DNA sequencer (Applied Biosystems 3130, Thermo Fischer Scientific), and manually edited to a 216 bp sequence and uploaded to GenBank acc. no. OQ834433. Twenty homologous *H. gingivalis* sequences were available in GenBank. A 216 bp-multiple sequence alignment was built with BioEdit software v. 7.2.5 [9]. The final dataset included 21 *H. gingivalis* sequences and 7 sequences from other genera. The most appropriate nucleotide substitution model (T92 + G + I) was selected by jModelTest2 [10] and used as model input for the Maximum Likelihood phylogenetic inference by MEGA v.11 [11]. The statistical robustness and reliability of the branching order were confirmed with bootstrap analysis using 1000 reiterations. Analysis of the obtained sequence showed a similarity ranging from 85.1 to 100.0% to 20 homologous sequences representing all the other *H. gingivalis* isolates available in the GenBank database. The current isolate was added to the already known isolates with a subsequent cluster analysis resulting in four clusters, the same four cluster as already identified by Nadler et al. [7]. The current isolate was determined to belong to Lineage 1 (Fig. 3).

## Discussion and conclusions

*Halicephalobus gingivalis* has been found in several Nordic countries [1, 12, 13] but the current case is the first known report of the entity in Sweden. It is difficult to fully determine if this horse was infected in Sweden or before it was imported from Iceland due to lack of knowledge regarding the incubation period. However, reports of foals euthanised and subsequently diagnosed with verminous meningoencephalitis caused by *H. gingivalis*, which had shown clinical signs as early as at 18 days of age, suggests that the disease has a short incubation period [14, 15]. The young age of the foals could however have contributed to a lower immune response leading to quick dissemination of this parasite. Another study describes progression of clinical signs in two adult horses developing in days to weeks [16], indicating a relatively rapid progression of disease even in adult horses. The horse in the presented case lived for five years in Sweden, which supports the hypothesis that the horse was infected in Sweden. The worldwide distribution of the parasite with occurrence in most neighbouring countries to Sweden strengthens this theory.

The acute development of neurological signs in the presented case suggested a rapid dissemination of nematodes within the central nervous system. The kidney lesions showed a more prolonged disease progress. This would indicate a primary infection of the kidney with subsequent spreading to the central nervous system. It is however unknown what triggered the spreading of the parasite from kidneys to brain. Blood smears taken during routine clinical examination did not reveal the presence of the nematodes, however the histopathological findings of seemingly viable nematodes in blood vessels suggest a hematogenous dissemination, as implied by several other publications [2]. The horse developed haematuria one year prior to euthanasia which could have been associated with infestation of *H. gingivalis* but is less likely due to clinical response to antimicrobial therapy and subsequent time lapse before development of neurological symptoms.

Treatment of *H. gingivalis* infestation in horses is difficult and the prognosis is poor, even when the lesions are thought to be focal [17]. Successful treatment of horses with a periorbital or cutaneous granuloma were reported through surgical debulking, intraoperative lavage with ivermectin and postoperative administration of ivermectin [18, 19]. Pharmacological treatment of horses with clinical neurological disease is not likely to be successful due to the inability of therapeutical dosage of ivermectin, the drug of choice, to penetrate the blood-brain barrier of mammals. Moreover, there is a risk of ivermectin toxicosis if the horse has an impaired blood-brain barrier due to meningitis [20].

Equine infection with *H. gingivalis* is exceedingly rare with no previous reports in Sweden. With the occurrence of *H. gingivalis* in a horse suggestively infected in Sweden, the disease should be added to the list of differential diagnoses in cases of acute onset of neurological disease and for horses with haematuria. This should also be done in other countries even if no previous cases have been reported as the findings of the current case indicate that the geographical spread of this soil-borne nematode could be larger than previously thought. Infestations may hence occur in countries without any prior cases of *H. gingivalis* and even in other species than horses, including humans. Our phylogenetic study reveals that the first Swedish *H. gingivalis* isolate belonged to Lineage 1, which includes other isolates from Europe, North America, Japan, and Australia. Notably, the only two *H. gingivalis* sequences available from human cases reported in Australia and Germany [21, 22] are both classified by phylogenetic analysis as belonging to Lineage 1. Unfortunately, a still limited number of sequences are available on public databases hampering any hypothesis on a link between zoonotic potential and genetic lineages. However, sequence similarity data and phylogenetic analysis seem to confirm previous observations on the lack of correlation between geographical origin and genetics in *H. gingivalis*, based on 28 S rRNA gene data [23]. With the help of genetic analysis in future cases, the relation between genetic variation and geographical spread can be further elucidated, which would be beneficial not only to prevent equine cases but also for public health.

## Data Availability

The dataset analysed during the current study are available from the corresponding author on reasonable request.

## Abbreviations

EHV: Equine herpesvirus
EIV: Equine influenza virus
FFPE: Formalin-fixed paraffin embedded
HE: Haematoxylin and eosin
